# Weight Loss‐Dependent Changes in Body Composition and Bone Health in People With Obesity and Type 1 Diabetes Treated With Liraglutide, Semaglutide, or Tirzepatide

**DOI:** 10.1002/dmrr.70213

**Published:** 2026-08-05

**Authors:** Ebaa Al Ozairi, Mohammad Irshad, Jumana Alkandri, Anant Mashankar, Stuart R. Gray, Carel W. le Roux

**Affiliations:** ^1^ DAFNE Unit Clinical Care Research and Trials Unit Dasman Diabetes Institute Kuwait City Kuwait; ^2^ Al‐Amiri Hospital Ministry of Health Kuwait City Kuwait; ^3^ Diagnostic Imaging Center Dasman Diabetes Institute Kuwait City Kuwait; ^4^ School of Cardiovascular and Metabolic Health University of Glasgow Glasgow UK; ^5^ Diabetes Complications Research Centre University College Dublin Dublin UK; ^6^ School of Medicine Ulster University Coleraine UK

**Keywords:** body composition, bone health, dual GIP/GLP‐1RA, GLP‐1RA, obesity, T1D

## Abstract

**Aims:**

This study examined changes in body composition and bone mineral density (BMD) over 12 months in people with obesity and type 1 diabetes (T1D) treated with a GLP‐1 receptor agonist alone (GLP‐1RA) or a dual glucose‐dependent insulinotropic polypeptide (GIP/GLP‐1RA).

**Materials and Methods:**

This real‐world observational study included 70 people with obesity and T1D treated with GLP‐1RA alone or dual GIP/GLP‐1RA. Dual‐energy X‐ray absorptiometry (DEXA) was used to quantify regional and total fat mass, lean mass, bone mineral content (BMC) and BMD at baseline and after 12 months of treatment. Overall changes were quantified, and changes stratified by percentage weight loss using repeated measures analysis.

**Results:**

Over 12 months body weight (−6.33%; 95% CI: −7.92, −4.73; *p* < 0.001), HbA1c (−0.48%; 95% CI: −0.74, −0.22; *p* < 0.001), total tissue fat (−1.42%; 95% CI: −2.47, −0.36; *p* = 0.009), fat mass (−5.90%; 95% CI: −10.50, −1.57; *p* = 0.001), and lean mass (−1.75%; 95% CI: −3.50, −0.48; *p* = 0.005) decreased, while total BMC and BMD remained unchanged. Total lean mass loss was associated with body weight loss (*R* ^2^ = 0.36, *p* < 0.001).

**Conclusions:**

In people with obesity and T1D, 12‐month GLP‐1RA alone or dual GIP/GLP‐1RA therapy resulted in clinically meaningful weight loss, improved glycaemic control, preferential fat mass reduction, modest lean mass loss, and preservation of total BMD. People achieving > 10% weight loss derived the greatest metabolic benefit without compromising bone health, although the loss of lean mass was greater.

## 1 Introduction

The prevalence of obesity among people with type 1 diabetes (T1D) has increased substantially over recent decades [1]. This represents a major challenge, as obesity is associated with insulin resistance, higher insulin requirements, poorer glycaemic control, increased cardiometabolic risk, and diabetes complications (nephropathy, neuropathy and retinopathy) [2, 3, 4, 5]. Obesity amplifies cardiovascular risk of diabetes through insulin resistance, chronic inflammation, dyslipidemia, hypertension, endothelial dysfunction, and cardiac remodelling [6]. People with obesity who also develop excessive ectopic fat may have direct organ damage, such as in the case of peri‐renal fat contributing to nephropathy [7]. Excessive visceral and ectopic fat can also contribute to increased inflammation that may further contribute to increased risk of retinopathy and neuropathy, although the latter two complications of diabetes have a stronger association with hyperglycaemia [8]. Glucagon‐like peptide‐1 receptor agonists (GLP‐1RAs) are incretin based therapies, developed for the treatment of obesity and type 2 diabetes (T2D). GLP‐1RA alone reduces cardiovascular risk [9, 10].

The use of GLP‐1RA, in combination with glucose‐dependent insulinotropic polypeptide (GIP) agonisms in the case of tirzepatide, as a treatment for the disease of obesity in people with T1D is growing rapidly, with a recent systematic review and meta‐analysis reporting significant reductions in body weight, HbA1c, and total daily insulin dose, although gastrointestinal adverse effects were more frequent in people with obesity and T1D [11]. In addition, randomised controlled trials have demonstrated modest but consistent reductions in body weight and improved glycaemic outcomes with GLP‐1RA alone compared with placebo in people with obesity and T1D [12].

Despite this emerging evidence supporting weight loss and modest glycaemic benefits [12, 13, 14], substantial uncertainty remains regarding the effects of GLP‐1RA alone or dual GIP/GLP‐1RA therapy on body composition and skeletal health in people with T1D and obesity. Body weight alone is a crude measure that does not differentiate between fat, lean tissue, and skeletal mass. Reductions in total and visceral fat are metabolically advantageous, while loss of lean tissue, including skeletal muscle and bone, may have adverse consequences for physical function, resting energy expenditure, and long‐term metabolic health.

Data on the effects of GLP‐1RA alone or dual GIP/GLP‐1RA therapy on bone health are mixed [15, 16]. Meta‐analyses conducted in populations with T2D or obesity generally indicate no significant detrimental effects of GLP‐1RAs alone or dual GIP/GLP‐1RA on bone mineral density (BMD) at major skeletal sites, although modest changes in bone turnover markers have been reported [15]. In contrast, clinical narrative reviews have indicated potential reductions in BMD associated with GLP‐1RA alone across populations with and without diabetes [16].

In people with obesity and T1D, evidence regarding the skeletal effects of GLP‐1RA alone or dual GIP/GLP‐1RA is limited. Initial findings suggest that adjunctive exenatide did not adversely affect BMD despite weight loss in adults with obesity and T1D [17]. Overall, the interplay between the magnitude of weight loss, body composition changes, and musculoskeletal health remains insufficiently characterised in people with obesity and T1D.

Furthermore, the degree of weight loss achieved may differentially influence body composition and skeletal responses, underscoring the importance of examining weight‐loss thresholds when interpreting compositional changes in people with obesity and T1D. Therefore, this real‐world observational study examined 12‐month changes in body composition and BMD in people with obesity and T1D treated with GLP‐1RA alone or dual GIP/GLP‐1RA. Additionally, we explored whether changes in fat mass, lean mass, and bone outcomes differed according to the degree of weight loss achieved.

## 2 Materials and Methods

### 2.1 Study Design and Participants

This was a prospective observational study. The Institutional Review Board of the Dasman Diabetes Institute provided ethical approval (RA HM–2020–018), and the study was conducted in accordance with the principles of the Declaration of Helsinki. All participants were of Arab ethnicity and receiving routine clinical care at the Dasman Diabetes Institute. Adults aged ≥ 18 years with a documented diagnosis of obesity and type 1 diabetes (T1D), based on the American Diabetes Association (ADA) criteria, treated with GLP‐1RA alone or dual GIP/GLP‐1RA therapy as part of routine dose adjustment for normal eating (DAFNE) clinical management were eligible for inclusion. GLP‐1RA alone or dual GIP/GLP‐1RA therapies were developed for obesity and people with T1D are not excluded according to the European Medicine Agency licence for obesity and the licence of these medications for obesity in Kuwait. These DAFNE graduates initiated GLP‐1RA alone therapies such as liraglutide and semaglutide or dual GIP/GLP‐1RA such as tirzepatide as per the licence for the treatment of obesity. Treatment followed standard clinical protocols for obesity, including gradual dose escalation, to minimise side effects and improve tolerability.

In this study, a total of 70 people with T1D were eligible, of which majority received liraglutide (70%, *n* = 49), followed by semaglutide (18.6%, *n* = 13) and tirzepatide (11.4%, *n* = 8). Of the total liraglutide users, the majority received a dose of 1.8 mg (79.6%), followed by 3 mg (20.4%). Among semaglutide users, the majority received a doses of 0.25–0.5 mg (46.2%), followed by 1 mg (23.0%) and 1.7–2.4 mg (30.8%). Among tirzepatide users, the majority received a doses of 2.5–5 mg (37.5%), 7.5–10 mg (25.0%), and 12.5–15 mg (37.5%).

### 2.2 Data Collection

Demographic, clinical, and biochemical variables, including age, sex, body weight, and HbA1c, alongside adverse events and treatment discontinuation were extracted from electronic health records at baseline and after 12 months of follow‐up.

### 2.3 Dual‐Energy X‐Ray Absorptiometry

As part of routine practice, body composition and bone parameters were assessed annually using dual‐energy X‐ray absorptiometry (DXA) (Lunar iDXA; GE Healthcare, Madison, WI, USA), analysed with enCORE software (iDXA enCORE). DXA technology utilises differential attenuation of X‐rays at high (approximately 70 keV) and low (approximately 40 keV) photon energies to distinguish between bone mineral content, lean tissue, and fat mass. Whole‐body DXA scans were performed according to standardised manufacturer protocols. The DXA software analysis algorithms were used to quantify regional and total body fat mass, percentage body fat, lean mass, bone mineral content (BMC) and BMD. In the DEXA algorithm, fat mass was expressed as an absolute value (g), whereas tissue fat (%) represents the proportion of fat relative to total body weight. Regional fat distribution was assessed by measuring android and gynoid fat mass. Android fat was defined as fat accumulation in the abdominal region between the ribs and pelvis, with its lower boundary at the iliac crest, whereas gynoid fat was defined as fat distribution around the hips and upper thighs. Quality assurance and calibration procedures were performed regularly in accordance with manufacturer guidelines.

### 2.4 Statistical Analysis

Statistical analyses were performed using SPSS Statistics software version 29.0 (IBM Corp., Armonk, NY, USA). Continuous variables were presented as mean ± standard deviation (SD), while categorical variables were reported as frequencies and percentages, where appropriate. In the full cohort, changes in outcomes from baseline to 12‐month follow‐up were analysed using general linear model (GLM) repeated‐measures tests, adjusted for age and sex. The changes from the baseline to 12 months were converted into percentage changes and results were reported as percentage mean differences with corresponding 95% confidence intervals (CIs) estimated via linear mixed models, adjusted for age and sex. Observed power was calculated to estimate the sensitivity of the analysis in detecting statistically significant differences. An observed power value of ≥ 0.80 was considered indicative of adequate statistical power. Spearman correlations were also performed between weight loss and body composition outcomes. Data were further stratified into subgroups based on the % weight loss (< 5%, 5%–10% and > 10%) and GLM repeated measures and ANCOVA tests employed to compare changes within and between the subgroups, adjusted for age and sex. All statistical tests were two‐sided, and a *p*‐value < 0.05 was considered statistically significant.

## 3 Results

The study included 70 people with obesity and T1D, comprising 33 (47.1%) males and 37 (52.9%) females. The mean ± SD age of participants was 36.7 ± 9.7 years (range: 22.9–53.1 years). There was no difference in age between males and females (mean difference = 3.1 years, *p* = 0.18). Baseline BMI was 31.7 ± 4.4 kg/m^2^, whereas HbA1c was 8.3 ± 1.4%. Detailed baseline body composition, BMD, and BMC parameters are presented in Table 1.

**TABLE 1:** Primary information of the people with T1D using GLP‐1RA alone or dual GIP/GLP‐1RA (*N* = 70).

	Mean	SD
Male, *n* (%)	33	47.1
Female, *n* (%)	37	52.9
Age (years)	36.7	9.7
Body weight (kg)	85.8	16.1
Height (cm)	165.4	10.1
BMI (kg/m^2^)	31.7	4.4
HbA1c (%)	8.3	1.4
Tissue fat arm (%)	40.6	10.0
Tissue fat legs (%)	42.8	9.4
Tissue fat trunk (%)	41.8	8.0
Tissue fat android (%)	42.7	8.8
Tissue fat gynoid (%)	44.4	8.5
Tissue fat total (%)	41.4	7.2
Tissue arms (g)	9273.3	2058.1
Tissue legs (g)	27,041.5	5194.7
Tissue trunk (g)	40,425.0	9539.5
Tissue total (g)	80,964.8	15,638.8
Fat mass arms (g)	3684.7	1030.9
Fat mass legs (g)	11,573.9	3429.2
Fat mass trunk (g)	17,471.0	6353.0
Fat mass android (g)	2685.8	1216.9
Fat mass gynoid (g)	5810.3	1669.9
Fat mass total (g)	33,625.9	9310.9
Lean mass arms (g)	5588.6	1840.4
Lean mass legs (g)	15,470.2	3858.4
Lean mass trunk (g)	23,097.3	4597.1
Lean mass android (g)	3414.1	747.1
Lean mass gynoid (g)	7228.5	1771.6
Lean mass total (g)	47,423.5	10,252.6
BMC total (g)	2396.9	435.0
BMD total (g/cm^2^)	1.16	0.10

Abbreviations: BMC, Bone mineral content; BMD, bone mineral density.

The changes in variables over the 12 month study period are presented in Table 2. After 12 months, there were reductions in body weight (−6.33%; 95% CI: −7.92, −4.73; *p* < 0.001), HbA1c (−0.48%; 95% CI: −0.74, −0.22; *p* < 0.001), total fat mass (−5.90%; 95% CI: −10.50, −1.57; *p* = 0.001), fat mass in arms (−5.87%; 95% CI: −10.50, −1.38, *p* = 0.001), fat mass in the legs (−4.98%; 95% CI: −8.50, −1.12; *p* = 0.003), fat mass in the trunk (−6.39%; 95% CI: −11.50, −1.09; *p* = 0.002), total lean mass (−1.75%; 95% CI: −3.50, −0.48; *p* = 0.005), lean mass in the arms (−4.52%; 95% CI: −6.50, −2.41; *p* < 0.001), lean mass in the trunk (−1.85%; 95% CI: −3.50, −0.28; *p* = 0.016) and lean mass in the gynoid (−2.32%; 95% CI: −3.50, −0.85; *p* = 0.003) (Table 2). BMD and BMC remained unchanged (all *p* > 0.05).

**TABLE 2:** Change in body composition in people with T1D after 12 months (*N* = 70).

	Percentage change	Absolute change^a^	*p* value^a^	Observed power^b^
	% (95% CI)	Mean (95% CI)		
Body weight	−6.33 (−7.92, −4.73)	−5.58 (−7.07, −4.10)	< 0.001	0.99
HbA1c	−0.48 (−0.74, −0.22)	−0.48 (−0.74, −0.22)	< 0.001	0.95
Tissue fat arm	−0.70 (−1.50, 0.32)	−0.70 (−1.72, 0.32)	0.179	0.27
Tissue fat legs	−1.29 (−2.50, −0.47)	−1.29 (−2.11, −0.47)	0.002	0.87
Tissue fat trunk	−1.22 (−2.50, 0.42)	−1.22 (−2.86, 0.42)	0.135	0.32
Tissue fat android	−1.35 (−3.50, 0.41)	−1.35 (−3.12, 0.41)	0.130	0.33
Tissue fat gynoid	−1.09 (−2.50, −0.05)	−1.09 (−2.14, −0.049)	0.040	0.54
Tissue fat total	−1.42 (−2.50, −0.36)	−1.42 (−2.47, −0.36)	0.009	0.76
Tissue arms	−5.39 (−7.50, −3.11)	−538.5 (−761.2, −315.8)	< 0.001	1.00
Tissue legs	−3.24 (−5.50, −0.90)	−936.3 (−1579.9, −292.6)	0.006	0.81
Tissue trunk	−4.30 (−7.50, −1.62)	−1962.4 (−3056.2, −868.6)	0.001	0.94
Tissue total	−3.59 (−5.50, −1.73)	−3105.9 (−4692.6, −1519.2)	< 0.001	0.97
Fat mass arms	−5.87 (−10.50, −1.38)	−283.2 (−440.0, −126.5)	0.001	0.94
Fat mass legs	−4.98 (−8.50, −1.12)	−680.8 (−1110.1, −251.5)	0.003	0.87
Fat mass trunk	−6.39 (−11.50, −1.09)	−1451.3 (−2338.8, −563.9)	0.002	0.90
Fat mass android	−2.35 (−9.50, 4.78)	−149.3 (−329.1, 30.5)	0.102	0.37
Fat mass gynoid	−5.24 (−9.50, −0.97)	−361.9 (−603.5, −120.3)	0.004	0.83
Fat mass total	−5.90 (−10.50, −1.57)	−2403.2 (−3821.6, −984.8)	0.001	0.91
Lean mass arms	−4.52 (−6.50, −2.41)	−254.6 (−376.3, −132.9)	< 0.001	0.98
Lean mass legs	−0.25 (−2.50, 1.47)	−44.3 (−303.8, 215.2)	0.738	0.06
Lean mass trunk	−1.85 (−3.50, −0.28)	−407.7 (−741.9, −73.4)	0.016	0.68
Lean mass android	−1.24 (−3.50, 0.60)	−41.7 (−101.3, 17.8)	0.163	0.29
Lean mass gynoid	−2.32 (−3.50, −0.85)	−160.4 (−263.6, −57.2)	0.003	0.87
Lean mass total	−1.75 (−3.50, −0.48)	−787.4 (−1336.1, −238.7)	0.005	0.82
BMC total	−0.04 (−0.50, 0.46)	−1.45 (−12.84, 9.94)	0.800	0.06
BMD total	0.02 (−0.50, 0.50)	0.0001 (−0.005, 0.006)	0.958	0.05

Abbreviations: −ve, decreased; +ve, increased; BMC, Bone mineral content; BMD, bone mineral density.

^a^ GLM repeated measure tests adjusted for age and sex.

^b^ Observed power calculated using alpha = 0.05.

### 3.1 Changes Stratified by Percentage Weight Loss

The change in variables over 12 months, stratified by the % weight loss, are presented in Table 3. In people with < 5% weight loss (*n* = 32), there were no significant changes in body weight, HbA1c, fat mass, lean mass, BMC, or BMD. In people with 5%–10% weight loss (*n* = 17), there were reductions in body weight (−7.28%; 95% CI: −8.8, −6.45; *p* < 0.001), fat mass (−5.41%; 95% CI: −14.79, −3.97; *p* = 0.019), and HbA1c (−0.31%; 95% CI: −0.60, −0.02; *p* = 0.037), while total lean mass, BMC and BMD remained unchanged. In people with > 10% weight loss (*n* = 21), there were reductions in body weight (−14.42%; 95% CI: −15.94, −12.89; *p* < 0.001), fat mass (−19.0%; 95% CI: −27.84, −10.16; *p* < 0.001), total lean mass (−3.47%; 95% CI: −5.53, −1.42; *p* = 0.006) and HbA1c (−0. 53%; 95% CI: −0.83, −0.24; *p* = 0.001) while BMC and BMD remained unchanged. Comparing between the subgroups, significant differences were observed in the reductions in body weight (*p* < 0.001) and fat mass (*p* < 0.001), whereas no significant differences were found for HbA1c (*p* = 0.674), lean mass (*p* = 0.114), BMC (*p* = 0.206), or BMD (*p* = 0.40).

**TABLE 3:** Change in body composition stratified by the % body weight loss after 12 months.

	Weight loss < 5%		Weight loss 5%–10%		Weight loss > 10%		ANCOVA
	*n* = 32	*p*‐value^b^	*n* = 17	*p*‐value^b^	*n* = 21	*p*‐value^b^	*p*‐value^a^
Body weight (%)	−0.51 (−1.79, 0.76)	0.292	−7.28 (−8.08, −6.45)	< 0.001	−14.42 (−15.94, −12.89)	< 0.001	< 0.001
HbA1c (%)	−0.54 (−0.90, 0.01)	0.054	−0.31 (−0.60, −0.02)	0.037	−0.53 (−0.83, −0.24)	0.001	0.674
BMD total (%)	−0.28 (−1.05, 0.48)	0.436	0.52 (−0.47, 1.50)	0.255	0.09 (−0.77, 0.95)	0.833	0.400
Tissue fat total (%)	0.46 (−0.59, 1.52)	0.378	−1.13 (−3.21, 0.95)	0.263	−4.51 (−6.80, −2.23)	0.001	< 0.001
Fat mass total (%)	2.43 (−2.33, 7.19)	0.276	−5.41 (−14.79, 3.97)	0.019	−19.0 (−27.84, −10.16)	< 0.001	< 0.001
Lean mass total (%)	−0.37 (−2.15, 1.35)	0.666	−2.12 (−5.21, 0.83)	0.071	−3.47 (−5.53, −1.42)	0.006	0.114
BMC total (%)	−0.20 (−0.82, 0.43)	0.537	0.56 (−0.29, 1.42)	0.101	−0.66 (−1.44, 0.12)	0.162	0.106

Abbreviations: −ve, decreased; +ve increased; BMC, Bone mineral content; BMD, bone mineral density.

^a^ ANCOVA analysis adjusted for age and sex.

^b^ GLM repeated measure test adjusted for age and sex.

**FIGURE 1:**
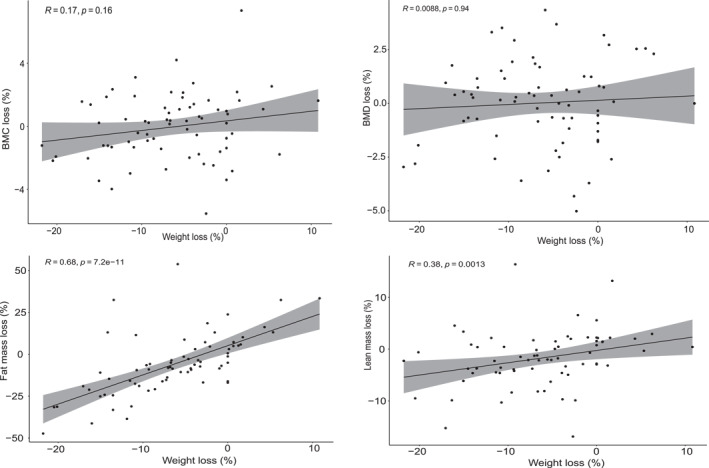
Spearman correlations were also performed between weight loss and body composition outcomes.

A Spearman's Rank Correlation for non‐normally distributed data showed monotonic relationships of percentage body weight loss with percentage lean mass (*R* ^2^ = 0.38, *p* = 0.001) and fat mass (*R* ^2^ = 0.68, *p* < 0.001) lost over the 12 month period (Figure 1).

No adverse effects, such as severe hypoglycaemia or DKA, were reported during the study period.

## 4 Discussion

In this real‐world observational study of people with obesity and T1D treated with GLP‐1RA alone or dual GIP/GLP‐1RA, clinically meaningful weight loss was accompanied by improvements in glycaemic control and favourable reductions in total and regional fat mass. These changes occurred alongside modest reductions in lean mass, while total BMC and BMD remained unchanged over 12 months. Importantly, the magnitude of body composition changes was strongly dependent on the degree of weight loss achieved. Individuals achieving > 10% weight loss experienced the greatest reductions in adiposity, with no change in BMD, with lean mass only declining in people losing > 10% body weight.

A key observation, of the current study, was the reduction in fat mass, particularly central, trunk, and android adiposity, with weight loss. These findings are consistent with prior studies in T2D and obesity, where GLP‐1RAs substantially reduce visceral and truncal fat depots [18, 19]. When looking at data stratified by the percentage of weight loss, individuals with minimal weight loss (< 5%) exhibited little change in body composition, with no changes in body fat despite small reductions in total body weight. In contract in individuals achieving > 10% weight loss, there were clear and clinically significant reductions in body fat but also in lean mass.

A meta‐analysis confirmed that the majority of clinical trials, in people with obesity and T2D, of GLP‐1RAs alone and dual GIP/GLP‐1RA report significant reductions in fat mass along with smaller declines in lean mass [20, 21]. To our knowledge, this is one of the first studies that confirmed this in people with T1D. Whilst there are clear and established benefits of the loss of body fat, importantly, the clinical implications of lean mass loss require nuanced interpretation. Absolute reductions in lean mass during weight loss do not necessarily imply adverse functional outcomes, particularly when the proportion of lean mass relative to total body weight is preserved or improved [22]. On the other hand, reductions in lean mass may possibly negatively affect metabolic rate, physical function, and bone integrity, particularly in frail or older individuals. The potential consequences of this loss in lean mass require further study.

Reassuringly, the overall preservation of total BMD was observed across all weight‐loss categories. Importantly, even among individuals achieving ≥ 10% weight loss, no significant deterioration in total BMD or BMC was observed. This contrasts with the substantial bone loss frequently reported following caloric restriction or bariatric surgery [23, 24] and suggests that GLP‐1RA alone or dual GIP/GLP‐1RA induced weight loss may be neutral on skeletal health. Experimental and clinical studies have proposed direct actions of GLP‐1RA on bone metabolism, including modulation of osteoblast and osteoclast activity, as well as indirect benefits mediated through improved glycaemic control, reduced inflammation, and decreased insulin resistance [25]. These mechanisms may partly explain the preservation of bone mass observed in this cohort.

Taken together, our results support the use of GLP‐1RA alone or dual GIP/GLP‐1RA to treat the disease of obesity in adults with T1D. The observed reductions in central fat mass and preservation of bone health, alongside reduction in HbA1c, are clinically meaningful and address key concerns related to long‐term cardiometabolic and skeletal outcomes in this population. However, the modest and weight loss dependent reductions in lean mass emphasise the need for comprehensive strategies to preserve lean mass when obesity medicines are used.

Several limitations should be acknowledged. The observational design precludes causal inference, and the absence of control group (neither treated with GLP‐1RA alone nor treated with dual GIP/GLP‐1RA) limits definitive attribution of observed changes to the intervention. Sample sizes within individual weight‐loss strata were relatively small, potentially reducing statistical power for some outcomes. In addition, 12 months may not be long enough to detect changes in BMD. The lack of significant change in total body BMD should not be interpreted as definitive evidence of stability for hip and spine BMD because BMD in these regions may decline even without a measurable change in total body BMD. Our data appear to suggest that BMD and BMC changed in opposite directions among those with the greatest weight loss. This may be explained by changes in body size and tissue thickness that may artificially alter bone area. This may also be a limitation of DXA measurements in the setting of large weight change. Additionally, data on dietary intake, physical activity, and changes in insulin dosing were unavailable and may have influenced body composition outcomes. Finally, collection of adverse events was limited to collecting severe hypoglycaemia and DKA, and other events such as gastrointestinal symptoms were not captured. This may limit the safety assessment.

In conclusion, in people with obesity and T1D, 12 months of GLP‐1RA alone or dual GIP/GLP‐1RA therapy was associated with significant weight loss and improvements in glycaemic control. As weight loss increased from < 5%, 5%–10%, and > 10%, so did the magnitude of improvement in body fat distribution. Declines in lean mass were observed only in those losing > 10% body weight, while bone mineral density was maintained. These findings provide important reassurance regarding skeletal safety, but cautions regarding lean mass loss, while highlighting the potential role of GLP‐1RA alone or dual GIP/GLP‐1RA therapy in addressing obesity‐related metabolic risk in T1D. Future randomised controlled trials with larger samples and longer follow‐up are warranted to confirm these findings.

## Author Contributions

Concept and design: Ebaa Al Ozairi and Carel W. le Roux. Data curation: Mohammad Irshad, Jumana Alkandri, and Anant Mashankar. Administrative and technical or material support: Ebaa Al Ozairi. Formal analysis, interpretation, and original draft writing: Ebaa Al Ozairi, Mohammad Irshad, Carel W. le Roux, and Stuart R, Gray. All authors critically reviewed and edited the manuscript, verified the underlying data, and approved the final version of the manuscript for publication.

## Funding

The manpower was funded by the Kuwait Foundation of Advancement of Science (KFAS) and the Ministry of Health (MOH), Kuwait. The funding agency did not influence the study design, data analysis, interpretation, or report preparation.

## Conflicts of Interest

The authors meet the criteria for authorship as recommended by the International Committee of Medical Journal Editors (ICMJE) and did not receive payment related to the development of this manuscript. Carel W. le Roux has received personal fees from Boehringer Ingelheim, Eli Lilly, GI Dynamics, Gila Pharmaceuticals, Herbalife, Johnson & Johnson, Keyron, Novo Nordisk, and Zealand Pharma outside the submitted work. Carel W. le Roux has received research funding from the MRC, NIHR, Jon Moulton Charitable Foundation, Fractyl, Gila, Randox and Novo Nordisk. Carel W. le Roux is a shareholder in the Beyond BMI clinic, which provides clinical obesity care.

## Data Availability

In adherence with the Dasman Diabetes Institute Policy on Transparency and Publication of Clinical Study Data, scientific and medical researchers can request access to clinical study data after publication of the manuscript.
